# Digital tool assessment for the community management of patients with pulmonary tuberculosis in Yiwu city, China: evidence from real world data in 2020

**DOI:** 10.3389/fpubh.2023.1320904

**Published:** 2024-01-08

**Authors:** Lin Zhou, Yuli Zhou, Yunfang Ding, Ying Peng, Wei Wang, Bin Chen, Shuiying Gong, Kui Liu, Xuanjun Dong

**Affiliations:** ^1^Department of Tuberculosis Control and Prevention, Zhejiang Provincial Center for Disease Control and Prevention, Hangzhou, China; ^2^The First People’s Hospital of Hangzhou Lin’an District, Hangzhou, China; ^3^Department of Tuberculosis Control and Prevention, Yiwu Municipal Center for Disease Control and Prevention, Jinhua, China

**Keywords:** tuberculosis, digital tool, community management, medication adherence, treatment outcome

## Abstract

**Background:**

High-quality medication compliance is critical for the cure of pulmonary tuberculosis (PTB); however, the implementation of directly observed treatment (DOT) under direct interview still faces huge difficulties. Assessment of the effect of digital tool during community management has not been performed in eastern China.

**Methods:**

All drug-sensitive PTB cases notified in Yiwu city from June to December 2020 were divided into the routine group and digital tool group based on patients’ willingness. The variables influencing the on-time completion level of home visits, medication adherence and treatment outcomes were estimated.

**Results:**

A total of 599 eligible patients were enrolled, with 268 participating in the routine group and 331 using a digital tool. Most participants were men (*n* = 357, 59.6%), and nearly all were new cases (*n* = 563, 94.0%). Participants’ mean age was 44.22 ± 20.32 years. There were significant differences in age, diagnostic type, and source of patients between the two groups. During the study period, the digital tool group had a higher on-time completion rate of home visits (91.5% vs. 82.5%) and medication adherence rate (94.3% vs. 89.6%) than the routine group, whereas there was no significant difference in the treatment success rate between the two groups (91.2% vs. 86.8%). Multivariate logistic regression analysis demonstrated that the digital tool group showed a more positive function in the on-time completion status of home visits, with an adjusted odds ratio of 0.41 (95% confidence interval: 0.25–0.70).

**Conclusion:**

Digital tools can be employed to improve the on-time completion rate of home visits in Yiwu city. Further large-scale studies that use digital tools for community management are warranted.

## 1 Introduction

Tuberculosis (TB) is a global problem that seriously endangers public health and is one of the top 10 causes of death worldwide (1). China is one of 30 countries with a high burden of TB worldwide. According to the World Health Organization’s Global TB Report released in 2022, the absolute number of TB in China accounts for 8.5% of the total high-burden countries, and 7.4% of the global total. Moreover, there are 780,000 new patients, ranking third in the world (2). The key to TB control is to find and cure the source of active cases to the greatest extent, while the core is to standardize the treatment throughout the whole process. Patients’ treatment compliance also directly affects the final treatment outcome.

Considering the long course of TB treatment and the possible combination of drugs, adverse reactions are prone to occur during the treatment phase, which seriously affects patient compliance with medication (3). In addition, the progress of curing TB with low-quality medication compliance can lead to serious consequences such as treatment failure, disease recurrence, and drug resistance (4). Many factors such as education, marital status, health insurance, and residence status affect medication compliance among patients with TB, and community management is one of the most important (5). Wherein, the presence of regular home-visiting by health workers had a significant impact on patients’ compliance. To improve the treatment compliance of patients with tuberculosis, the World Health Organization recommended directly observed chemotherapy (DOT) provided by medical staff or supervisors trained by medical staff (6). China also mainly implemented the DOT strategy in the past few decades. Increased studies indicate that the DOT strategy is not completely suitable for different regions or patients (7). There were considerable difficulties in the implementation of DOT, and a substantial number of patients are self-medicating, which affects treatment outcomes (8–10).

Currently, the supervision and medication administration for patients with pulmonary tuberculosis (PTB) has been included in the national basic public health service project as part of the “health management of tuberculosis patients” (11). Community doctors completed first home visits to patients, trained family supervisors, and regularly visit patients until the end of their treatment course. However, during the follow-up process, patients’ medication status cannot be obtained in real-time, which is not conducive to patient management (12). With modern awareness of personal privacy protection, traditional management models have been resisted, and the demand for promoting supervision models that meet individual needs has become increasingly strong (13). With the continuous emergence and maturity of new technologies, digital tools such as mobile applications, WeChat, and electronic pillboxes are increasingly used to assist in patients’ community management (14, 15). Available evidence has shown that digital tools improve patients’ medication adherence and provide feedback on the results to medical staff in a timely manner. Timely feedback can help identify patients with poor compliance and timely interventions can be provided (16). However, evidence from the assessment of digital tools used for the community management of TB remains limited.

In Zhejiang province, Yiwu city was the first city to apply digital tools to provide community management services for patients with TB in June 2020. This study evaluates the effect of digital tools used in the community management of PTB cases in Yiwu city and provides a basis for the feasibility of promoting the application of digital tools in the future.

## 2 Materials and methods

### 2.1 Location

**Figure 1:**
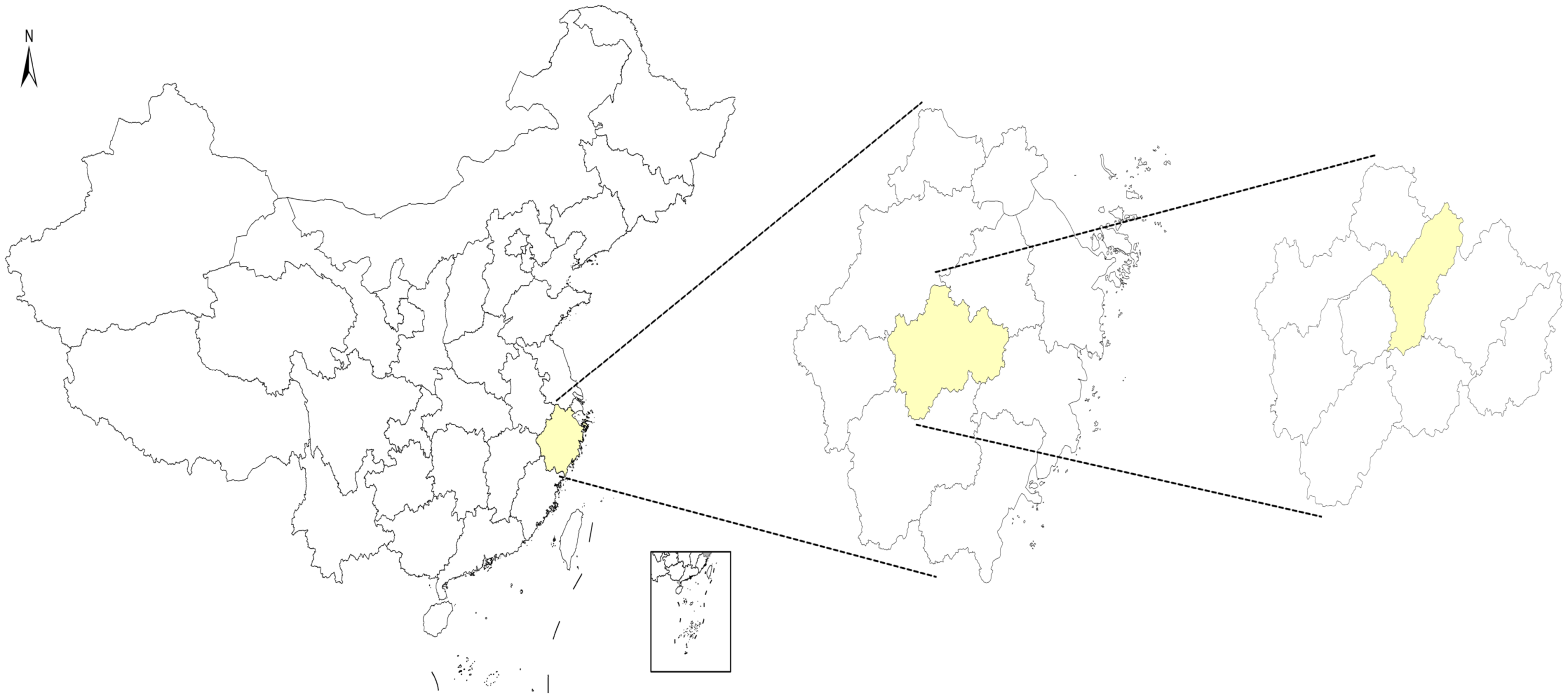
The location of Yiwu city.

Yiwu city is located in the middle of Zhejiang province, with 14 towns (streets) under its jurisdiction. The permanent population is about 1.88 million. Yiwu city had 880 newly registered patients with PTB in 2020, with a reported incidence rate of 66.74/100,000. The location is presented in Figure 1.

### 2.2 Participants and data collection

This was a retrospective cohort study based on real-world data, including 599 patients with drug-sensitive PTB who were notified in Yiwu city from June to December 2020 as the research participants. The 599 cases were divided into a routine group and a digital tool group according to their own willingness, with 268 cases and 331 cases, respectively. The permanent residences of all participants were in Yiwu city. The data of the routine group were mainly from the “tuberculosis information management system” (a national special system for tuberculosis management), while all the data of the digital tool group were mainly from the management system provided by the digital tool.

### 2.3 Definition

All recruited participants were patients with drug-sensitive PTB. The routine group included patients who adopted routine care, including supervisors from their families or the patients themselves. The medical staff would conduct their first home visit and regular telephone follow-up to improve adherence. During home visits, some routine work such as determining a supervisor, evaluating the living environment of patients, and providing health education for patients and their family members would be performed. The digital tool group included patients who were using a digital tool to improve medication compliance except routine management. The digital tool was developed by the Beijing SINOVO Power Technology Company (China) and customized by the Yiwu Municipal Center for Disease Control and Prevention based on local conditions. The digital tool provided two options to patients—the reminder application or reminder smart pillbox—while one management application along with a management website were provided for medical staff to monitor patients’ medication adherence. A detailed description of tool utilization was introduced previously (17, 18).

On-time completion rate of home visits (%) = number of patients completed on-time for home visit/number of patients included in this study × 100%.

Medication adherence rate (%) = number of patients with good medication adherence/number of patients included in this study × 100%. The definition of good medication adherence is that the medication rate ≥90% during the entire treatment course of the patient.

Treatment success rate (%) = number of patients treated successfully/number of patients included in this study × 100%. The treatment outcomes of curing and completing the course of treatment were defined as successful treatment, whereas treatment failure, death, loss to follow-up, and other outcomes were defined as unsuccessful treatment.

These indicators implied timely health education and sufficient medication guidance, ensuring possibility of high medication adherence, thereby avoiding the occurrence of drug resistance and improving the ultimate cure rate.

### 2.4 Statistical analysis

Data analysis was performed using R software (version 3.4.3). The quantitative data were described as “case (percentage, %)” and conducted chi-squared tests. When chi-squared tests were not appropriate, Fisher’s exact probability was used. All influencing factors were included in the multivariate logistics regression model. Significance was set at *p* < 0.05.

## 3 Results

### 3.1 Participants’ general characteristics

From June to December 2020, 599 eligible patients were enrolled in Yiwu city, with 268 participating in the routine group and 331 using digital tool. A total of 357 (59.6%) participants were men, and 563 (94.0%) were new cases. Participants were mainly farmers and migrant workers. There were no significant differences in the distribution of sex, occupation, or TB type between two groups. The proportion of patients younger than 60 years-old was higher in the digital tool group (82.2%) than in the routine group (64.2%). The proportion of negative cases was higher in the digital tool group (57.7%) than in the routine group (47.8%). The proportion of local cases in the digital tool group (35.0%) was lower than in the routine group (53.0%). Significant differences were observed in the distribution of age, TB cases classification, and population type (Table 1).

**Table 1:** Participants’ general characteristics.

Variable	Total	Routine group	Digital tool group	*p*-value
	(*N* = 599)	(*n* = 268)	(*n* = 331)	
Age (years)				<0.001
≤60	444 (74.1%)	172 (64.2%)	272 (82.2%)	
>60	155 (25.9%)	96 (35.8%)	59 (17.8%)	
Mean ± SD	44.22 ± 20.32	48.94 ± 21.11	40.40 ± 18.84	
Sex				0.584
Male	357 (59.6%)	163 (60.8%)	194 (58.6%)	
Female	242 (40.4%)	105 (39.2%)	137 (41.4%)	
Occupation				0.164
Farmer/migrant worker	391 (65.3%)	183 (68.3%)	208 (62.8%)	
Others	208 (34.7%)	85 (31.7%)	123 (37.2%)	
TB type				0.156
New cases	563 (94.0%)	256 (95.5%)	307 (92.7%)	
Previously treated cases	36 (6.0%)	12 (4.5%)	24 (7.3%)	
TB cases classification				0.015
Negative	319 (53.3%)	128 (47.8%)	191 (57.7%)	
Positive	280 (46.7%)	140 (52.2%)	140 (42.3%)	
Population type				<0.001
Local	258 (43.1%)	142 (53.0%)	116 (35.0%)	
Migrant	341 (56.9%)	126 (47.0%)	215 (65.0%)	

### 3.2 The difference between variable indicators among two groups

Three indicators were selected to evaluate the differences between the two management modes. During the observation period, the on-time completion rate of first home visit in the digital tool group was 91.5% higher than 82.5% in the routine group. The medication adherence rate in the digital tool group was 94.3% higher than 89.6% in the routine group. The treatment success rate in the digital tool group was 91.2% higher than 86.9% in the routine group but no significant difference was observed (Table 2).

**Table 2:** Supervision and management of study participants.

	Total	Routine group	Digital tool group	*p*-value
	(*N* = 599)	(*n* = 268)	(*n* = 331)	
On-time completion of home visit				0.001
Complete	524 (87.5%)	221 (82.5%)	303 (91.5%)	
Incomplete	75 (12.5%)	47 (17.5%)	28 (8.5%)	
Medication adherence				0.033
Good	552 (91.2%)	240 (89.6%)	312 (94.3%)	
Poor	47 (7.8%)	28 (10.4%)	19 (5.7%)	
Treatment outcome				0.090
Success	535 (89.3%)	233 (86.9%)	302 (91.2%)	
Failure	64 (10.7%)	35 (13.1%)	29 (8.8%)	

### 3.3 Logistic regression analysis for different indicators

We retrospectively analyzed the association between independent variables (routine and digital tool groups) and dependent variables (compliant of home visit, good medication adherence, and favorable treatment outcome) after adjusting for covariates (age, sex, occupation, TB type, TB cases classification and population type). Compared with routine group, the digital tool group could improve the on-time completion status of home visit, and the difference was significant [adjusted OR: 0.41, (95% CI: 0.25–0.70), Table 3], while the association with medication adherence and favorable treatment outcome was non-significant. Multifactor analysis results showed that patients aged 60 years or older were less likely to have good medication adherence as compared to their counterparts [adjusted OR: 4.07, (95% CI: 1.78–9.33), Table 4] and a favorable outcome [adjusted OR: 3.74, (95% CI: 1.87–7.47), Table 5]. Univariate analysis showed a correlation between population type and treatment outcomes, but multivariate analysis did not show a correlation (Table 5).

**Table 3:** Univariate and multivariate analysis of factors associated with on-time completion of home visit.

Variable	Univariate analysis		Multivariate analysis	
	Adjusted OR (95% CI)	*p*-value	Adjusted OR (95% CI)	*p*-value
*Group*				
Routine group	1.00	0.001	1.00	0.001
Digital tool group	0.44 (0.26–0.72)		0.41 (0.25–0.70)	
*Age (years)*				
≤60	1.00	0.312	1.00	0.228
>60	1.31 (0.77–2.23)		1.52 (0.77–3.02)	
*Sex*				
Male	1.00	0.669	1.00	0.726
Female	1.11 (0.68–1.82)		1.09 (0.66–1.81)	
*Occupation*				
Farmer/migrant worker	1.00	0.200	1.00	0.179
Others	1.38 (0.84–2.26)		1.43 (0.85–2.93)	
*TB type*				
New cases	1.00	0.792	1.00	0.974
Previously treated cases	0.87 (0.30–2.52)		0.98 (0.33–2.93)	
*TB cases classification*				
Negative	1.00	0.212	1.00	0.160
Positive	0.73 (0.45–1.20)		0.69 (0.41–1.16)	
Population type				
Local	1.00	0.940	1.00	0.279
Migrant	1.02 (0.63–0.66)		1.41 (0.76–2.62)	

**Table 4:** Univariate and multivariate analysis of factors associated with medication adherence.

Variable	Univariate analysis		Multivariate analysis	
	Adjusted OR (95% CI)	*p*-value	Adjusted OR (95% CI)	*p*-value
*Group*				
Routine group	1.00	0.522	1.00	0.110
Digital tool group	0.52 (0.29–0.96)		0.60 (0.32–1.12)	
*Age (years)*				
≤60	1.00	<0.001	1.00	<0.001
>60	3.05 (1.67–5.58)		4.07 (1.78–9.33)	
*Sex*				
Male	1.00	0.219	1.00	0.247
Female	0.67 (0.36–1.27)		0.68 (0.36–1.31)	
*Occupation*				
Farmer/migrant worker	1.00	0.674	1.00	0.844
Others	0.87 (0.46–1.65)		1.07 (0.55–2.09)	
*TB type*				
New cases	1.00	0.455	1.00	0.634
Previously treated cases	1.51 (0.51–4.47)		1.31 (0.43–4.04)	
*TB cases classification*				
Negative	1.00	0.222	1.00	0.155
Positive	1.45 (0.80–2.64)		0.68 (0.40–1.16)	
*Population type*				
Local	1.00	0.251	1.00	0.264
Migrant	0.71 (0.39–1.28)		1.60 (0.70–3.62)	

**Table 5:** Univariate and multivariate analysis of factors associated with treatment outcomes.

Variable	Univariate analysis		Multivariate analysis	
	Adjusted OR (95% CI)	*p*-value	Adjusted OR (95% CI)	*p*-value
*Group*				
Routine group	1.00	0.092	1.00	0.650
Digital tool group	0.64 (0.38–1.08)		0.88 (0.51–1.53)	
*Age (years)*				
≤60	1.00	<0.001	1.00	<0.001
>60	4.50 (2.63–7.67)		3.74 (1.87–7.47)	
*Sex*				
Male	1.00	0.192	1.00	0.190
Female	0.69 (0.40–1.20)		0.68 (0.39–1.21)	
*Occupation*				
Farmer/migrant worker	1.00	0.734	1.00	0.441
Others	0.91 (0.52–1.58)		1.26 (0.70–2.27)	
*TB type*				
New cases	1.00	0.523	1.00	0.801
Previously treated cases	1.38 (0.52–3.68)		1.14 (0.41–3.20)	
*TB cases classification*				
Negative	1.00	0.179	1.00	0.676
Positive	1.43 (0.85–2.41)		1.13 (0.65–1.95)	
*Population type*				
Local	1.00	<0.001	1.00	0.425
Migrant	0.36 (0.21–0.61)		0.75 (0.38–1.51)	

## 4 Discussion

The level of patient medication compliance directly affects the treatment effect and outcome of TB, and it is crucial for the control of the TB epidemic (19). Some studies have showed that the first home visit of medical staff was an important factor influencing the treatment compliance of patients with tuberculosis. The first home visit and medication adherence are important indicators for evaluating management quality. Therefore, improving these indicators is of great value. An increasing number of electronic tools are being used to improve these indicators (20). However, an evaluation of how much they can be improved compared to traditional methods has not yet been conducted.

No digital tool can be applied to all patients, as they are affected by age, occupation, education level, disease classification, and treatment course (21, 22). Compared to older adult patients, young and middle-aged patients are more willing to accept digital tool for acquiring disease-related knowledge and medication management (23). This was confirmed by our study that patients in the digital tool group were younger than those in the routine group. Pathogenic negative patients were more willing to accept digital tool, which is inconsistent with Mengxian’s research (18). This could be related to education level and disease cognition. Pathogenic negative patients have a higher degree of awareness of TB, and they were diagnosed and detected early. Compared to local populations, the migrant population is more willing to accept digital tools, which could be related to the younger age of migrant patients with TB.

The on-time completion rate of first home visit (91.5%) and medication compliance (94.3%) of the digital tool group were higher than those of routine group (82.5% and 89.6%, respectively), which is consistent with prior results (24). The first home visit is an important part of the community management of patients with TB. If patients use digital tool for community management, more comprehensive trainings and app itself could provide sufficient health education to patients and family medication supervisors. Therefore, the on-time completion rate of first home visit is important for medication compliance. This study suggests that the application of digital tool for community management can effectively improve the completion of first home visit and medication compliance of patients.

The key to applying a digital tool is to perform classified interventions and precisely manage patients. This study found that 5.7% of patients who used the digital tool still took medication irregularly and required a classified intervention. Although the international standard no longer insists on DOT management for all patients with TB, it still clearly stipulates that medical personnel should not only formulate the correct treatment plan but also assess patients’ compliance with treatment management and address patient irregularities in a timely manner (25). Although the application of smart tool has enabled patients and treatment managers to provide real-time, two-way feedback on patients’ medication information, medical staff should not pay too much attention to the data provided by smart tool. Care and social support run through the entire patient management process and strengthen the community management of patients who discontinue medication or treatment. Whether medical personnel implement intervention measures is crucial for improving treatment compliance and affecting treatment outcomes.

Age is an important influencing factor of medication compliance and treatment outcomes, and the drug compliance and treatment success rates of patients aged 60 years or older are lower than those of patients in other age groups, which is consistent with past research results (26). This could be related to the lack of awareness of the disease and the importance of regular medication in older adult patients with PTB, the common comorbidities in older adult patients with PTB, or the negative attitude toward the cure of the disease and their inactive treatment in older adult patients with PTB. This study suggests that in community management of patients with TB, it is necessary to focus on patients with TB aged 60 years or older.

### 4.1 Limitations

This study has some limitations. First, the sample size was small, and the representativeness of the results could have been biased. In addition, this study was conducted during the COVID-19 pandemic; thus, possible effects including insufficient medical services and inconvenient medical treatment for patients might affect the results.

## 5 Conclusion

Digital tool utilization can effectively improve the completion of first home visit and medication compliance of patients in Yiwu city. Further large-scale studies that use a digital tool for community management are warranted.

## Data availability statement

The original contributions presented in the study are included in the article/supplementary material, further inquiries can be directed to the corresponding authors.

## Ethics statement

The studies involving humans were approved by the Ethics Committee of the Zhejiang Provincial Center for Disease Control and Prevention (2022-032-01). The studies were conducted in accordance with the local legislation and institutional requirements. The ethics committee/institutional review board waived the requirement of written informed consent for participation from the participants or the participants’ legal guardians/next of kin because given that only TB surveillance data were used, the requirement for informed consent was waived.

## Author contributions

LZ: Formal analysis, Writing – original draft, Data curation. YZ: Writing – review & editing. YD: Data curation, Writing – review & editing. YP: Writing – review & editing, Formal analysis. WW: Writing – review & editing, Data curation. BC: Writing – review & editing, Formal analysis. SG: Writing – review & editing, Data curation. KL: Writing – review & editing, Data curation, Formal analysis. XD: Writing – review & editing, Data curation.
